# Therapeutic hypothermia initiated within 6 hours of birth is associated with reduced brain injury on MR biomarkers in mild hypoxic-ischaemic encephalopathy: a non-randomised cohort study

**DOI:** 10.1136/archdischild-2018-316040

**Published:** 2018-11-13

**Authors:** Paolo Montaldo, Peter J Lally, Vânia Oliveira, Ravi Swamy, Josephine Mendoza, Gaurav Atreja, Ujwal Kariholu, Vijayakumar Shivamurthappa, Natasha Liow, Justinas Teiserskas, Russell Pryce, Aung Soe, Seetha Shankaran, Sudhin Thayyil

**Affiliations:** 1 Centre for Perinatal Neuroscience, Imperial College London, London, UK; 2 Neonatal Unit, Imperial Healthcare NHS Trust, London, UK; 3 Oliver Fisher Neonatal Unit, Medway NHS Hospital Foundation Trust, Gillingham, UK; 4 Perinatal-Neonatal Medicine, Wayne State University, Detroit, Michigan, USA

**Keywords:** neonatology, neurology

## Abstract

**Objective:**

To examine the effect of therapeutic hypothermia on MR biomarkers and neurodevelopmental outcomes in babies with mild hypoxic-ischaemic encephalopathy (HIE).

**Design:**

Non-randomised cohort study.

**Setting:**

Eight tertiary neonatal units in the UK and the USA.

**Patients:**

47 babies with mild HIE on NICHD neurological examination performed within 6 hours after birth.

**Interventions:**

Whole-body cooling for 72 hours (n=32) or usual care (n=15; of these 5 were cooled for <12 hours).

**Main outcome measures:**

MRI and MR spectroscopy (MRS) within 2 weeks after birth, and a neurodevelopmental outcome assessment at 2 years.

**Results:**

The baseline characteristics in both groups were similar except for lower 10 min Apgar scores (p=0.02) in the cooled babies. Despite this, the mean (SD) thalamic NAA/Cr (1.4 (0.1) vs 1.6 (0.2); p<0.001) and NAA/Cho (0.67 (0.08) vs 0.89 (0.11); p<0.001) ratios from MRS were significantly higher in the cooled group. Cooled babies had lower white matter injury scores than non-cooled babies (p=0.02). Four (27%) non-cooled babies with mild HIE developed seizures after 6 hours of age, while none of the cooled babies developed seizures (p=0.008). Neurodevelopmental outcomes at 2 years were available in 40 (85%) of the babies. Adverse outcomes were seen in 2 (14.3%) non-cooled babies, and none of the cooled babies (p=0.09).

**Conclusions:**

Therapeutic hypothermia may have a neuroprotective effect in babies with mild HIE, as demonstrated by improved MRS biomarkers and reduced white matter injury on MRI. This may warrant further evaluation in adequately powered randomised controlled trials.

**What is already known on this topic?**

- Therapeutic hypothermia for 72 hours reduces brain injury on neonatal MRI and improves later neurodevelopmental outcomes after moderate or severe hypoxic-ischaemic encephalopathy (HIE).
- Despite a lack of evidence, many cooling centres in the UK and in other high-income countries routinely cool babies with mild HIE.

**What this study adds?**

- Whole-body cooling initiated within 6 hours of birth and continued for 72 hours reduces cerebral metabolite perturbations and white matter brain injury seen on MRI.
- A small proportion of the non-cooled babies with mild HIE may develop seizures after 6 hours of age and progress to moderate HIE.

## Introduction

Several major cooling trials in the past decade provide conclusive evidence for the safety and efficacy of cooling therapy in improving survival without disability after moderate or severe hypoxic-ischaemic encephalopathy (HIE) in high-income countries.^1^ These trials excluded babies with mild HIE, and hence the optimal neuroprotective strategies for these babies remain unclear. Although adverse neurodevelopmental outcomes at 2 years or more are seen in up to 25% of infants with mild HIE, pooled data from clinical trials involving 117 babies fail to show any benefits of therapeutic hypothermia in mild HIE.^2^ Despite this uncertainty, cooling therapy has crept into the routine management of babies with mild HIE.^3^

MRI and MR spectroscopy (MRS) biomarkers can accurately quantify brain injury and assess the treatment effects of neuroprotective therapies in HIE with greater power than clinical outcome measures, and hence require reduced sample sizes.^4^ Unlike moderate or severe HIE, babies with mild HIE often have white matter injury, and deep brain nuclei injury is often not visible on conventional MRI.^5^ However, the thalamus is one of the most metabolically active areas in the fetal brain, and hence some degree of apoptosis is invariably seen in the thalami in preclinical models even with milder ischaemic insults.^6^ Thalamic proton MRS is exquisitely sensitive to these changes.

The aim of this observational study was to examine the effect of whole-body hypothermia on cerebral MR biomarkers and neurological outcomes at 2 years after mild HIE.

## Methods

We identified babies with mild HIE who were recruited as part of the large international, prospective multicentre Magnetic Resonance Biomarkers in Neonatal Encephalopathy (MARBLE) study between January 2013 and June 2016.^7^

Consecutive full-term (≥36 weeks) infants admitted to the neonatal intensive care unit with mild HIE following a perinatal hypoxic event were included in this substudy of MARBLE. We accepted any of the following as evidence of perinatal asphyxia: metabolic acidosis on cord/baby blood gas within 1 hour of birth; an acute intrapartum event (eg, cord prolapse, abruption, antepartum bleed); a 10 min Apgar score ≤5; or assisted ventilation initiated at birth and continued ≥10 min.

Babies who fulfilled these criteria underwent a standardised neurological examination within 6 hours of birth using the NICHD Neonatal Research Network trial of hypothermia, by a certified examiner, with extended criteria to include mild HIE.^8 9^ We defined mild HIE as infants with ≥2 abnormal categories but with no evidence of moderate or severe HIE (defined as moderate and/or severe abnormality in ≥3 categories).

All babies had amplitude integrated electroencephalography (aEEG) for at least the first 6 hours after birth. We classified the aEEG background activity as normal (the upper margin of band of aEEG activity >10 μV and the lower margin >5 μV), moderately abnormal amplitude (the upper margin of band of aEEG activity >10 μV and the lower margin <5 μV), or severely abnormal amplitude (the upper margin of the band of aEEG activity <10 μV and lower margin <5 μV). Infants were divided into two groups: those who received 72 hours of therapeutic hypothermia and those who did not receive any hypothermia or had hypothermia for ≤12 hours. The decision to provide therapeutic hypothermia was based on the attending clinician’s preference.

### MR acquisition and analysis

MR scans were performed between 4 and 14 days of age on a 3T scanner (Philips, GE or Siemens) with harmonised protocols. We used MRS phantoms with known metabolite concentrations to cross-calibrate the scanners from each site prior to recruitment. All MR data were analysed centrally masked to the clinical details and outcome data (online supplementary eTable 1). The MR protocol and data analysis plan has been published previously.^7^

10.1136/archdischild-2018-316040.supp1 Supplementary file 1

### Neurodevelopmental assessment

At 2 years of age, we performed a detailed neurological examination using Bayley-III. In cases where Bayley-III was not available, the British Association of Perinatal Medicine/Royal College of Paediatrics and Child Health working group classification was used instead.^10^

We considered any disability at 18–24 months of age as defined by the NICHD Neonatal Research Network as an adverse outcome.^11^ Mild disability was defined by a cognitive score of 70–84 alone, or a cognitive score ≥85 and Gross Motor Function Classification System (GMFCS) level 1 or 2; a seizure disorder (without antiepileptic medication); or hearing deficit with ability to follow commands without amplification. Moderate disability was classified as: cognitive score from 70 to 84 and GMFCS level 2; active seizure disorder (receiving antiepileptic medication); or hearing deficit with the ability to follow commands after amplification. Finally, severe disability was defined as: cognitive score <70; GMFCS levels 3–5; blindness; or hearing impairment with inability to follow commands in spite of amplification.

### Statistical analysis

Statistical analysis involved using a two-sided Student’s t-test for parametric continuous variables and a Mann-Whitney U test for continuous non-parametric variables. Categorical variables were compared using a Χ^2^ test or Fisher’s exact test. Analyses were performed with SPSS Statistics V.24 (IBM) and a p value of <0.05 was considered statistically significant.

## Results

A total of 47 babies with mild HIE were included in the study. Of these, 15 (32%) were not cooled (n=10) or were cooled for less than 12 hours (n=5, median duration of cooling 3 hours IQR 3–10), and 32 (68%) had 72 hours of cooling. The baseline characteristics of cooled and non-cooled babies were similar apart from significantly lower 10 min Apgar scores in the cooled babies (p=0.02, table 1). The hospital stay was significantly longer for the cooled babies (p=0.04, table 1). Eight (25%) of the cooled babies and 1 (6%) of the non-cooled babies had a moderately abnormal aEEG within 6 hours of age (p=0.2).

**Table 1:** Baseline characteristics and neonatal course of the cooled and non-cooled babies

	No cooling (n=15)	72 hours of cooling (n=32)
Antenatal history		
Maternal diabetes	2 (13)	0
Maternal hypertension	1 (6)	3
Thyroid disease	0	0
Reduced fetal movements	2 (13)	2 (6)
CTG bradycardia	2 (13)	10 (31)
Late decelerations	1 (6)	2 (6)
Variable decelerations	1 (6)	1 (3)
Prolonged rupture of membranes*	2 (13)	6 (18)
Birth and resuscitation		
Gestational age at birth (weeks)	39.0 (2.0)	40 (1.2)
Birth weight (kg)	3.2 (0.4)	3.6 (0.4)
Cord pH	7.1 [0.5]	6.9 [0.2]
Cord BE	−9.2 [6.8]	−14.0 [6.7]
Temperature admission	36.0 (0.6)	36.0 (0.8)
Apgar score (1 min)	4 [4]	2 [3]
Apgar score (5 min)	6 [2]	5 [2]
Apgar score (10 min)	8 [2]†	6 [3]†
Neonatal course		
Abnormal aEEG	1 (6)	8 (25)
Invasive ventilation	7 (46)	22 (68)
Non-invasive ventilation	1 (6)	3 (9)
Hypotension requiring inotropes	2 (13)	5 (15)
Abnormal clotting	1 (6)	6 (19)
Decreased urine output	0	3 (9)
Maximum creatinine levels during the first 72 hours of age	80 (22)	78 (17)
Maximum CRP levels during the first 72 hours of age	22 (44)	23 (23)
Blood culture positivity	0	1 (3)
Seizures after 6 hours of age	4 (27)	0
Postnatal age at MRI (days)	6.5 [3.0]	7.0 [4.0]
Hospital stay (days)	5.5 [6.2]†	8.0 [2.0]†

Data are mean (SD), n (%), median [IQR].

*Suspected or confirmed rupture of membranes for more than 24 hours.

†P<0.5 (Mann-Whitney U test).

aEEG, amplitude integrated electroencephalography; CRP, C-reactive protein; CTG, cardiotocography; BE, Base Excess; MRI, magnetic resonance imaging

Four (8.5%) babies with mild HIE and normal aEEG developed seizures after 6 hours of age (online supplementary eTable 2). Of these, one baby (case 4) was subsequently cooled for 72 hours, starting from 10 hours of age.

On conventional MRI, 29 out of the 47 (61%) infants had abnormal white matter. The white matter lesion scores in the non-cooled babies (mean 1.3, SD 0.7) were significantly higher than in the fully cooled babies (mean 0.5, SD 0.6, p=0.01) (table 2). Abnormal white matter was seen in 50% (16/32) of the cooled babies and 87% (13/15) of the non-cooled babies (p=0.02) (online supplementary eFigure 1).

10.1136/archdischild-2018-316040.supp2 Supplementary file 2

**Table 2:** MRI and MR spectroscopy in cooled and non-cooled groups with mild encephalopathy

Conventional MRI	No cooling (n=15)	72 hours of cooling (n=32)
Basal ganglia and thalami, n (%)		
Normal	14 (93.3)	30 (93.8)
1	1 (6.7)	2 (6.3)
2	0	0
3	0	0
Posterior limb of internal capsule, n (%)		
Normal	15 (100)	32 (100)
1	0	0
2	0	0
3	0	0
White matter, n (%)		
Normal	2 (13.3)	16 (50)
1	9 (60)	13 (40.6)
2	4 (26.7)	3 (9.4)
3	0	0
Cortex, n (%)		
Normal	12 (80)	29 (90.6)
1	3 (20)	3 (9.4)
2	0	0
3	0	0

**Table:** 

MR spectroscopy	No cooling (n=14)	72 hours of cooling (n=26)
Lac/NAA	0.16 (0.06)	0.12 (0.03)
NAA/Cho	0.67 (0.08)*	0.89 (0.11)*
NAA/Cr	1.42 (0.11)*	1.64 (0.18)*

Data are number of patients or mean (SD).

*P<0.001 (Student’s t-test).

Cho, total choline; Cr, total creatinine; Lac, threonine+lactate; NAA, N-acetylaspartate+N-acetylaspartylglutamate.

Only three infants had injury to the deep brain nuclei, two of whom had been cooled for 72 hours, while one was not cooled. There was no difference in injury scores between the two groups in the deep nuclei or cortex.

**Figure 1:**
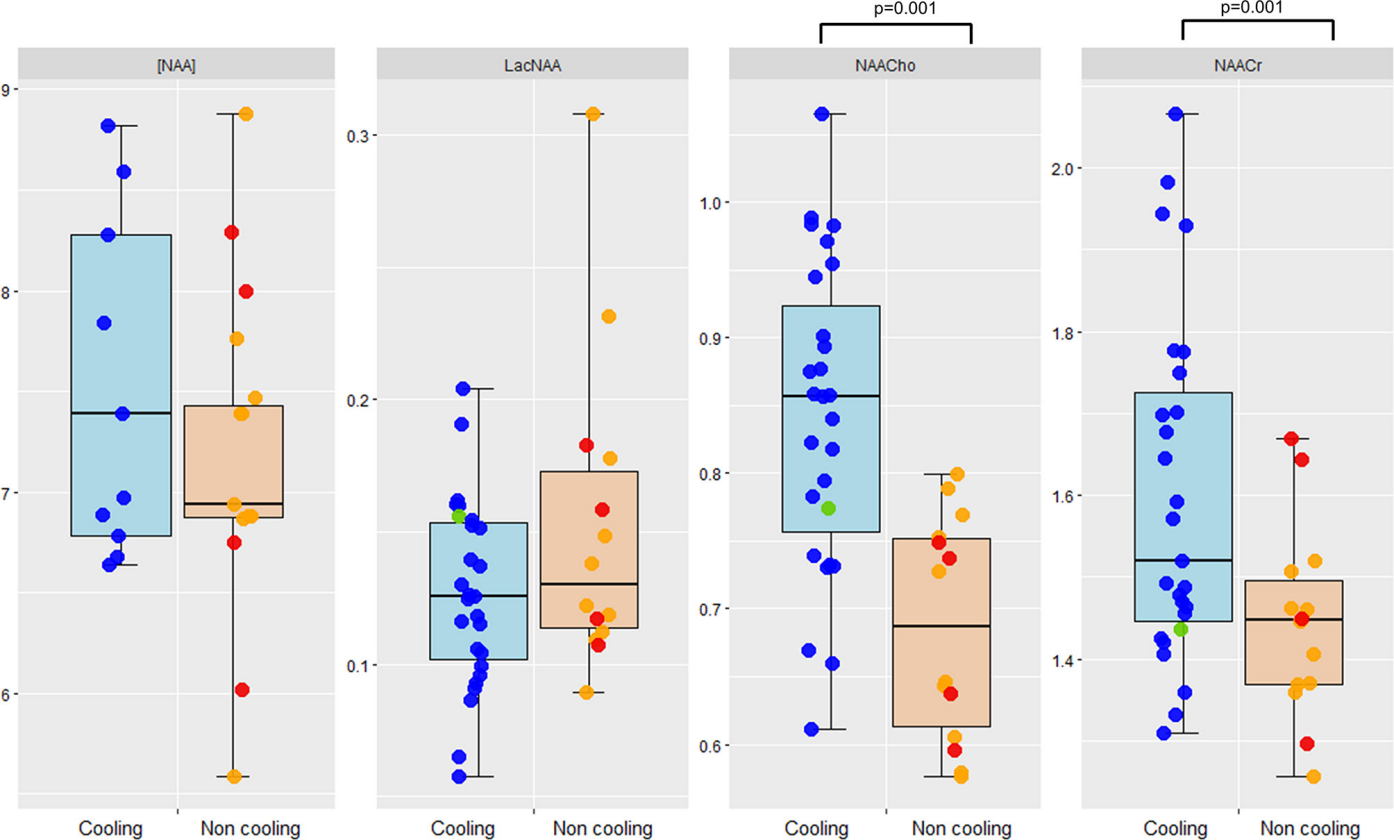
Dot plots (media, IQR) for MR spectroscopy measures in the cooled and non-cooled babies with mild encephalopathy. Blue dots indicate babies cooled for 72 hours, red dots indicate babies cooled for less than 24 hours, green dots indicate babies cooled after 6 hours, and orange dots indicate non-cooled babies. Lac/NAA, threonine+lactate/NA; NAA/Cho, N-acetylaspartate+N-acetylaspartylglutamate/total choline; NAA/Cr, N-acetylaspartate+N-acetylaspartylglutamate/total creatinine.

Six babies (one non-cooled and five cooled) were excluded from the MRS analysis due to poor quality data, or loss of data during transfer from the scanner. From the remaining MRS data, the thalamic ratios of NAA/Cr and NAA/Cho (adjusted for gestational age) were significantly higher in cooled babies than non-cooled babies (figure 1). After the exclusion of those infants who progressed to moderate HIE, the differences in brain injury between cooled and non-cooled babies persisted, with thalamic ratios of NAA/Cr and NAA/Cho significantly higher in fully cooled babies and white matter injury scores significantly higher in the non-cooled group.

No significant differences were found in the thalamic ratios of Lac/NAA between the two groups (p=0.21) (figure 1). Only two babies with mild HIE, who did not undergo cooling, had an elevated (≥0.22) Lac/NAA ratio indicating a significant injury to the deep brain nuclei, and a high risk of adverse neurodevelopmental outcome.^12^ The Lac/NAA ratios were all below this threshold in the cooled babies.

**Figure 2:**
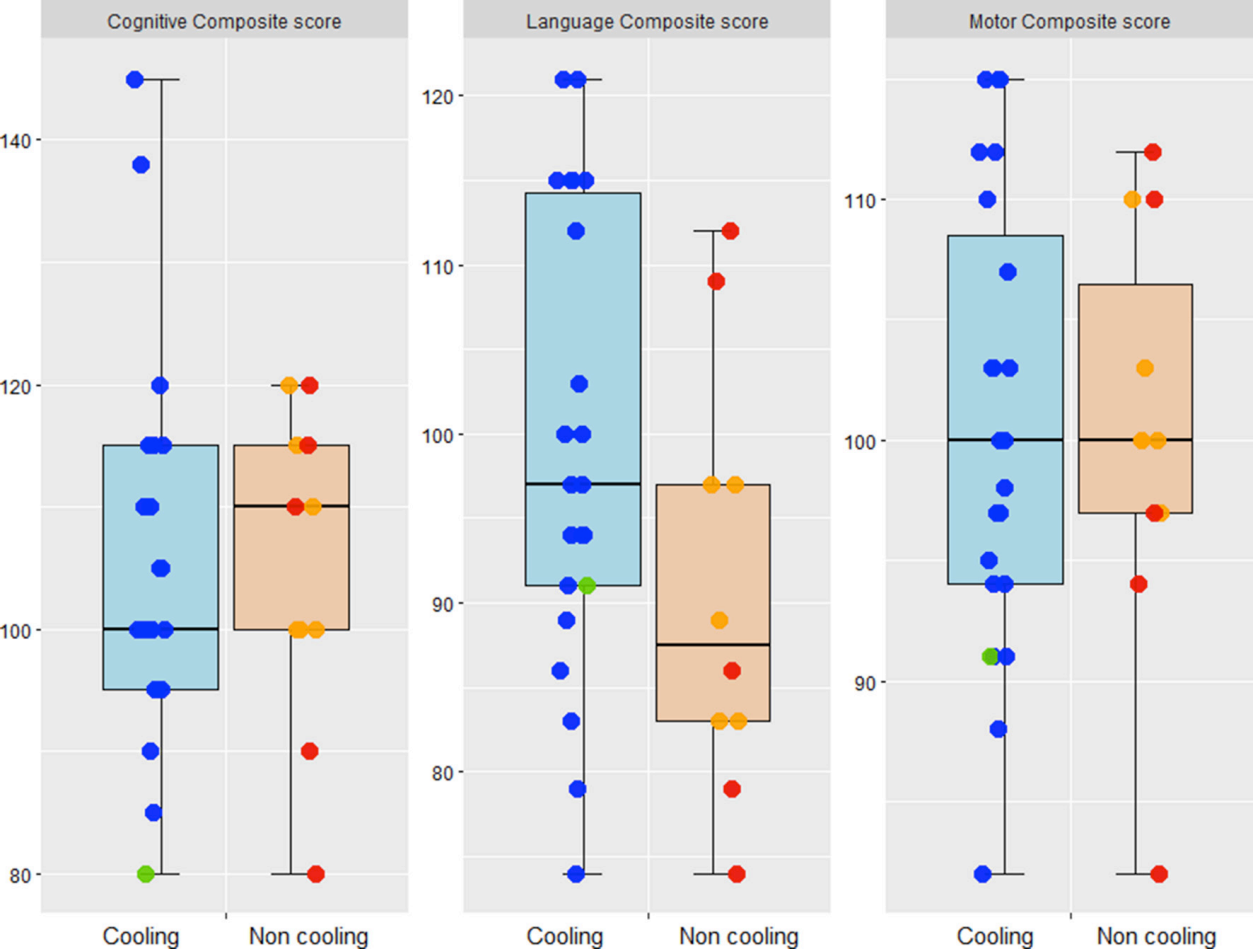
Dot plots (median, IQR) of the neurodevelopmental outcome scores in the cooled and non-cooled babies with mild encephalopathy. Blue dots indicate babies cooled for 72 hours, red dots indicate babies cooled for less than 24 hours, green dots indicate babies cooled after 6 hours, and orange dots indicate non-cooled babies.

A total of 40 (85%) infants had neurodevelopmental outcome assessments for analysis (table 3 and figure 2). There was no significant difference in terms of baseline characteristics between the children who were lost at the follow-up and the remaining ones. Two non-cooled infants had mild disability at 2 years. One developed seizures at 33 hours of age, and was later diagnosed with hyperekplexia, confirmed by a homozygous novel missense mutation SLC6A5 (case 3; online supplementary eTable 2). At the time of neurological assessment (18 months of age) there was mild motor disability without cerebral palsy and severe language delay. In the second case, the Bayley-III assessment (at 21 months of age) assigned a cognitive composite score of 80 (equivalent developmental age of 16 months), a language composite score of 74 (receptive communication equivalent developmental age of 16 months; expressive communication equivalent developmental age of 15 months) and a motor composite score of 82 (fine motor equivalent developmental age of 17 months; gross motor equivalent developmental age of 16 months). The infant had right arm monoplegia (GMFCS=1).

**Table 3:** Neurodevelopmental outcomes at 2 years after mild encephalopathy

	No cooling (n=14)	72 hours of cooling (n=26)
Cognitive composite score	106 (13)	105 (15)
Language composite score	90 (12)	99 (25)
Motor composite score	100 (9)	100 (10)
Motor composite score <85	1 (7)	1 (3)
Language composite score <85	4	2 (8)
Cognitive composite score <85	1 (7)	1 (3)
Cerebral palsy	1 (7)	0

Data are number (%), mean (SD).

There was no statistically significant difference between the cooled and non-cooled groups.

## Discussion

In this multicentre prospective observational study on 47 babies with mild HIE at 6 hours of age, cooled babies had less white matter injury and improved thalamic metabolic indicators in comparison to non-cooled babies, despite having lower 10 min Apgar scores. Four of the 15 non-cooled babies developed seizures and progressed to moderate HIE after 6 hours of age. The differences in brain injury between cooled and non-cooled babies persisted even after excluding these babies. The mean cognitive, motor and language scores at 2 years were similar in the cooled and non-cooled babies. However, 2 out of the 14 (14.3%) cases in the non-cooling group had mild disability whereas none of the babies who were cooled had an adverse outcome at 2 years.

There has been only limited research so far describing the nature of injury in mild HIE and the effect of cooling therapy. Previous studies demonstrated that infants with mild HIE have predominately white matter injury whereas infants with moderate or severe HIE are more likely to have injury to the basal ganglia and thalami.^13 14^ This is likely to reflect different underlying disease mechanisms, since white matter injury is typically associated with subacute hypoxia, whereas injury to the deep nuclei is associated with acute profound asphyxia.^15^ However, in those studies none of the infants were cooled and therefore it was not possible to investigate the effect of cooling in mild HIE.

A previous study showed similar results with a lower incidence of brain injury on MRI in infants who were treated with whole-body cooling (4/13 (31%)) when compared with those who did not receive cooling (20/50 (40%)).^16^ In that study, the authors found that the majority of neonates with mild HIE had an abnormal signal in the cortex regardless of whether or not they were cooled. Of note, recent animal evidence suggests that therapeutic hypothermia preserves white matter when the preclinical model is subjected to mild hypoxia-ischaemia.^17^

In another retrospective study on 89 encephalopathic babies who received cooling therapy, Walsh *et al* 18 reported a similar incidence of watershed injury in infants who had mild HIE (18/48 (36%)) when compared with those with moderate (11/32 (32%)) or severe HIE (3/6 (50%)). Only 2 (4%) babies with mild HIE had abnormal signal in thalamic or basal ganglia in that study. Our data confirm these observations. We did not find any significant thalamic injury on the conventional MRI of babies with mild HIE, although some degree of white matter injury was seen in 50% (16/32) of the cooled babies and 87% (13/15) of the non-cooled babies. In contrast, we found evidence of improved thalamic MRS markers (higher NAA/Cr and NAA/Cho) in cooled babies with mild HIE, although Lac/NAA was normal in all babies except two. However, in spite of these differences in MRS, the neurodevelopmental outcome at 18–24 months was not different between the cooled and non-cooled infants. Although neurodevelopmental outcome is currently the gold standard of measuring efficacy in cooling therapy, our study was not powered to examine/detect any differences in the clinical outcomes.

Preclinical studies have observed neuroprotection with 24–48 hours of cooling, while very short periods or very long periods of cooling are not neuroprotective.^19^ Although the numbers were small, our study suggests that cooling for less than 24 hours is unlikely to be beneficial in mild HIE, as there was no difference in the metabolite profile of non-cooled and partially cooled babies. We previously demonstrated that there was MRI evidence of residual brain injury in 50% of the babies with mild HIE who were cooled for less than 24 hours, and 20% of them subsequently had adverse outcomes.^20^ Whether cooling for 24 or 48 hours is as good or better than cooling for 72 hours in mild HIE is not known.

The development of seizures and progression of mild HIE to moderate or severe HIE after 6 hours of age, and thus missing the therapeutic window for cooling, is a major concern for clinicians. Only 4 (8.5%) babies with mild HIE progressed to moderate HIE after 6 hours in our study. Of these, only one had an adverse neurodevelopmental outcome at 2 years and was subsequently diagnosed with hyperekplexia. Gagne-Loranger *et al* 16 reported progression of mild HIE to moderate in 6% of babies (12% in the non-cooled group and 3% in the cooled group), which is consistent with the data from the Prospective Research in Mild Encephalopathy study,^21^ where a standardised neurological examination was performed. On the other hand, the Infant Cooling Evaluation (ICE) trial reported progression to moderate NE in 50% of babies with mild HIE. The neurological examination was not standardised in the ICE trial, and it is possible that babies were misclassified as mild HIE at the initial examination.^22^

Currently, the use of therapeutic hypothermia in babies with mild HIE is creeping into clinical practice despite a lack of evidence. In a recent survey, including 54 of the 68 UK cooling centres, 61% of the centres provided cooling for babies with mild HIE for the full 72 hours irrespective of any clinical improvement.^3^ Separately, recent surveys have reported a similar therapeutic drift, with almost half of the cooled babies in Australia and 15% of the cooled babies from the Children’s Hospitals Neonatal Consortium in USA, lacking the features of moderate or severe HIE at initiation of cooling therapy.^23 24^

Although our study suggests a possible therapeutic benefit of cooling in mild HIE, caution is needed before drawing definitive conclusions, and a clinical decision to cool should not be taken lightly. Cooling therapy requires intensive care, often transfer to specialist centres and separation from parents, in addition to increased healthcare costs. One newborn piglet study has suggested cooling of the healthy brain may induce apoptosis,^25^ although this has not been replicated in other preclinical models.^26^ Given this lack of evidence and widespread variation in practice, there is an urgent need to investigate the impact of whole-body cooling in mild HIE infants in adequately powered randomised controlled trials.

This study has strengths and limitations. This is a small observational study, and hence no definitive conclusions on the therapeutic benefits of cooling can be made. Despite these, significant differences were identified in the metabolite peak area ratios of the cooled and non-cooled babies, suggesting that further evaluation of hypothermic neuroprotection in mild HIE is warranted. Our study was not powered to examine the clinical outcomes. Given the low event rates of adverse outcomes after mild HIE, thousands of babies would be required to detect improvements in neurodevelopmental outcomes. Study strengths include a robust prospective multicentre design, examinations performed by certified examiners, MRI and MRS analysis by a blinded central reader and scanner harmonisation prior to the start of the study.

## Conclusion

Our study demonstrates that babies with mild HIE have less white matter and metabolic brain injury after therapeutic hypothermia, suggesting that cooling may have a neuroprotective effect in these infants.
