# Supplementary material for: Therapeutic hypothermia initiated within 6 hours of birth is associated with reduced brain injury on MR biomarkers in mild hypoxic-ischaemic encephalopathy: a non-randomised cohort study
Source: Arch Dis Child Fetal Neonatal Ed. 2018 Nov 13;104(5):F515–20. doi: 10.1136/archdischild-2018-316040 (PMC6788875; doi:10.1136/archdischild-2018-316040)

eTable 1. Conventional MRI scoring system

Basal ganglia and thalamic score
0= normal
1= mild (focal abnormal signal intensity)
2= moderate (multifocal abnormal signal intensity)
3= severe (widespread abnormal signal intensity)

Posterior limb of the internal capsule score
0= normal
1= equivocal (reduced or asymmetrical signal intensity)
2= loss (reversed or abnormal signal intensity bilaterally on T_1 and/or T_2-weighted sequences)

White matter score
0= normal
1= mild (exaggerated long T_1 and long T_2 in periventricular white matter only)
2= moderate (long T1 and long T2 extending out to subcortical white matter and/or focal punctate lesions or focal area of infarction)
3= severe widespread abnormalities including overt infarction, hemorrhage and long T_1 and long T_2. Infarction was classified as areas of excessive long T_1 and long T_2 with either loss of gray-white matter differentiation (usually within the first week) or exaggerated gray-white matter differentiation (after the first week). Optic radiations were scored abnormal if they demonstrated increased signal intensity on T_1-weighted images

Cortex score
Cortical involvement was scored as the presence of abnormal signal intensity, usually decreased T_1 or cortical highlighting. The sites documented included specifically the central sulcus, interhemispheric fissure and the insula
0= normal
1= mild (1-2 sites involved)
2= moderate (3 sites involved)
3= severe (more than 3 sites involved)

eTable 2. Clinical characteristics, MR results and neurodevelopmental outcomes of the mildly encephalopathic babies who progressed to moderate encephalopathy after six hours

			Case No 1	Case No 2	Case No 3	Case No 4
Delivery			Em LSCS[2]	SVD[1]	SVD[1]	Instrumental
Type of admission			Outborn	Inborn	Inborn	Inborn
Gestational age (weeks+days)			41+0	40+3	38+1	37+6
Birth Weight (g(centile))			3270(25-50)	4140(75-91)	4640(91-98)	2630(2-9)
Cardiotocography (CTG)			Other abnormality	Normal	Normal	Other abnormality
Arterial cord pH			7.0	7.0	7.0	7.0
Arterial cord base excess			NA	NA	-9.9	NA
pH on first blood gas			6.9	7.1	7.2	7.1
Apgar 1 minute			1	4	6	6
Apgar 5 minute			5	6	8	9
Apgar 10 minute			6	8	10	
Resuscitation at birth			Bag and mask	Bag and mask	Bag and mask	Oxygen
Complications at birth			None	Shoulder dystocia	Shoulder dystocia	Failure to progress
Inotropic support at admission			Yes	No	No	No
Neurological examination at admission* 0= normal 1= mild 2= moderate 3= severe	LOC[4]		1	1	1	1
	Spont. Activity		1	1	1	0
	Posture		0	1	1	1
	Tone		1	1	1	1
	Reflexes[5]	Moro	0	0	1	0
		Suck	0	0	0	0
	Auton. System[6]	Pupils	0	0	0	0
		Heart rate	0	0	0	0
		Respiration	0	0	0	0

Maternal Pyrexia		Yes	No	No	No
Prolonged rupture of membranes		No	No	No	Yes
Rectal temperature on admission (°C)		38.2	36.3	37.0	37.0
Age at start of active cooling (hours)		NA	NA	NA	12
Maximum CRP in first 72 hours of age(g/L) [7]		3	5	4	4
Blood/CSF positive culture		No	No	No	No
Seizure onset age, hours		12	7	33	10
MRI	Cortex	0	0	0	1
	Basal ganglia/thalamus	0	0	0	0
	White matter	2	1	1	1
	Posterior limb of the internal capsule	0	0	0	0
[8]Lac/NAA		0.29	0.12	0.11	0.15
Neurodevelopmental Outcome at 2 years		Unknown	Normal	Abnormal**	Normal
Cognitive composite score		NA	100	NA	80
Language composite score		NA	83	NA	91
Motor composite score		NA	100	NA	91
aEEG		Normal	Normal	Normal	Normal

[1]SVD=spontaneous vaginal delivery; [2]Em LSCS = Emergency lower segment caesarean section; [4]LOC= Level of consciousness; [5]Reflexes category include Moro and suck; [6]Autonomic System including pupils, respiration and heart rate. [7]CRP= C-reactive protein; [8]Lac/NAA: (lactate+threonine)/(N-acetylaspartate+N-acetylaspartylglutamate); *To classify as mild encephalopathy, babies had at least one abnormality in the neurological examination, but no evidence of moderate or severe encephalopathy (which requires at least 3 moderate or severe abnormalities)
**Only a detailed neurological examination was performed, without Bayley-III. This infant also had hyperekplexia.

eFigure 1. Typical example of white matter injury showing multiple focal punctate lesions (white arrows) on axial T_2-weighted (A), T_1-weighted and diffusion weighted MR images.

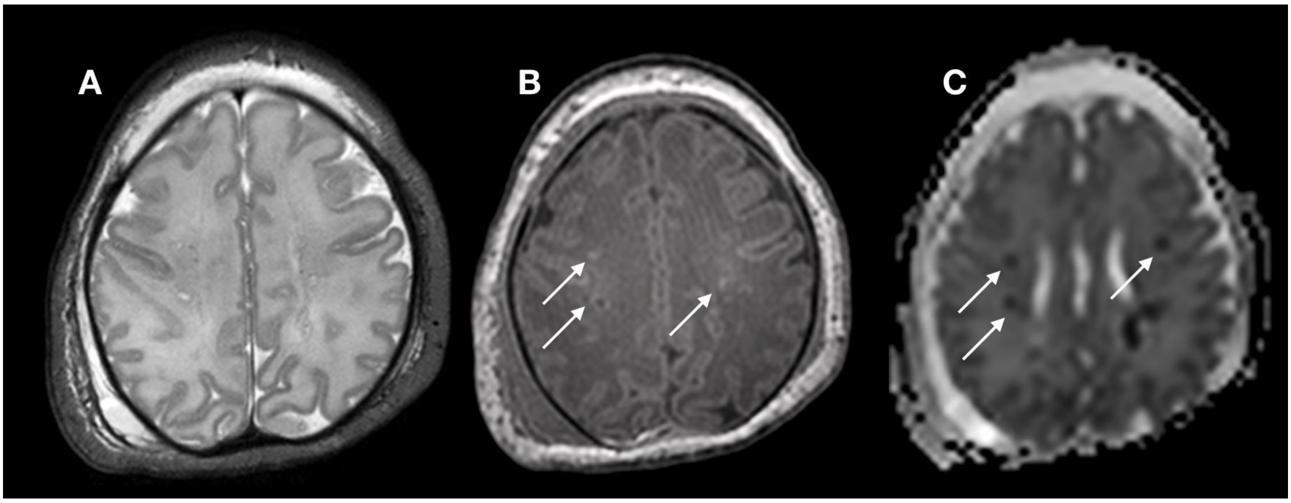

Supplement: Supplementary file 1 [file fetalneonatal-2018-316040supp001.pdf]
