# Supplementary figures and images for: Therapeutic hypothermia initiated within 6 hours of birth is associated with reduced brain injury on MR biomarkers in mild hypoxic-ischaemic encephalopathy: a non-randomised cohort study
Source: Arch Dis Child Fetal Neonatal Ed. 2018 Nov 13;104(5):F515–20. doi: 10.1136/archdischild-2018-316040 (PMC6788875; doi:10.1136/archdischild-2018-316040)

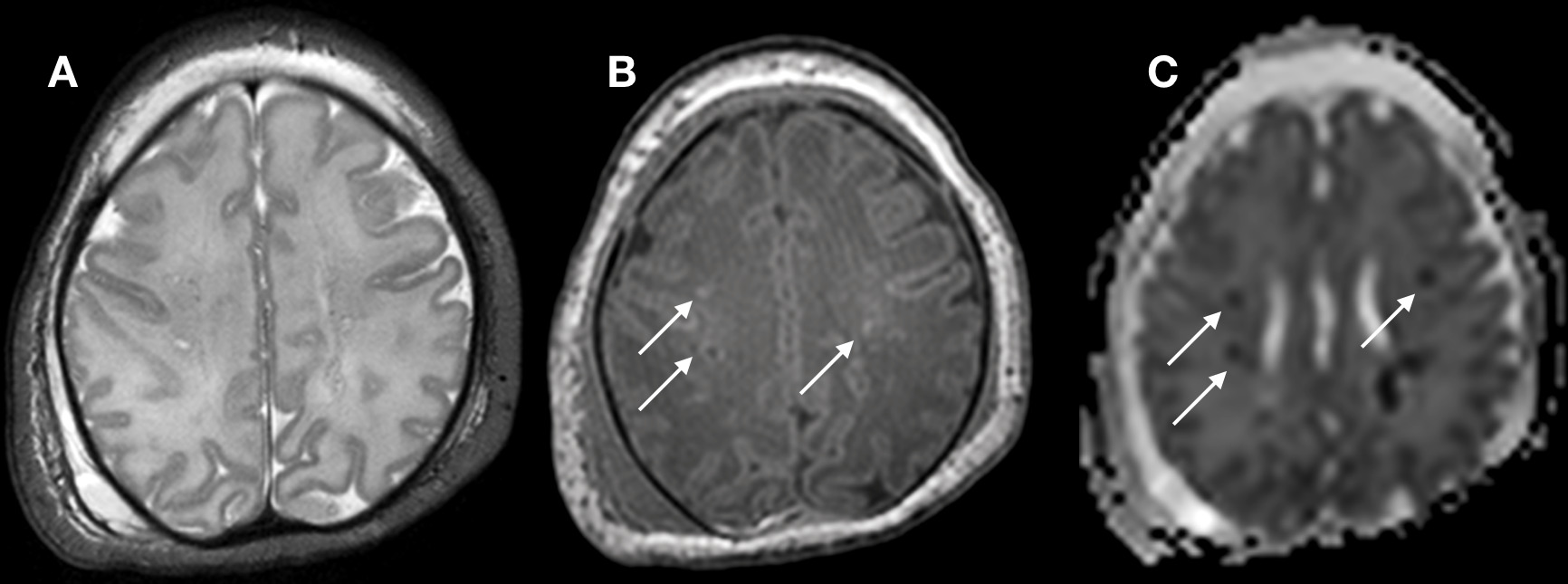

Supplement: Supplementary file 2 [file fetalneonatal-2018-316040supp002.jpg]
